# Could pre-diabetes be considered a clinical condition? opinions from an endocrinologist and a cardiologist

**DOI:** 10.1186/1758-5996-2-2

**Published:** 2010-01-15

**Authors:** Maria Eliane C Magalhães, Breno A Cavalcanti, Saulo Cavalcanti

**Affiliations:** 1 Division of Cardiology, State University of Rio de Janeiro, Rio de Janeiro, Brazil; 2 School of Medical Sciences of Minas Gerais, Belo Horizonte, Brazil; 3 Division of Endocrinology, School of Medical Sciences of Minas Gerais, Belo Horizonte, Brazil

## Abstract

The prevalence of pre-diabetes is increasing worldwide and may start 7 to 10 years before the clinical diagnosis of diabetes. In this stage the presence and accumulation of risk factors is common and already implies an increase in cardiovascular risk. Likewise, the onset of cardiovascular diseases (CVD), mainly coronary artery disease (CAD), peripheral vascular disease and cerebrovascular disease can also take place, all of which account for high rates of morbidity and mortality worldwide. Considering pre-diabetes as a clinical entity, non-pharmacological and pharmacological treatments are indicated with drugs which have shown clinical benefits related to reduction in morbidity and mortality. However, there is still need for new long-term studies to assess the real benefits of several new therapeutical approaches, as well as its cost-effectiveness.

## Introduction

The natural history of type 2 diabetes has been studied in many populations and it has been described as a relationship among a genetic susceptibility, obesity and physical inactivity.

Both obesity and physical inactivity result in insulin resistance states, which in turn will stress beta cell to increase insulin secretion Beta cell dysfunction - it is considered an important early pathophysiologic defect in diabetes type 2 and it is present since the pre-diabetes phase [1]. This fact had been confirmed in a study, which performed autopsies in normal individuals, glucose intolerant and patients with diabetes showed that even during pre-diabetes phase there is a loss of beta cell function up to 50% due apoptosis [2]. However according to Prof De Fronzo the loss of beta cell function could be even more, up to 50% [1].

Nowadays the treatment of type 2 diabetes must address the core of pathophisiologic defects of the disease which means that we have to treat both beta cell dysfunction and insulin resistance as soon as we have the diagnosis of diabetes, and perhaps as early as we have the diagnosis of glucose intolerance [1]. However, the slow increase in glyceamic levels - currently referred to as dysglicaemia - causes considerable delay in both diagnosis and consequently the beginning of the treatment. Nonetheless, during this phase, some underlying mechanisms common to micro and macrovascular complications, like endothelial dysfunction and oxidative stress, are already present [3]. Nowadays the continuous relationship between blood glucose and cardiovascular disease (mortality and morbidity) and the development of diabetes type 2 is a matter of concern and will be discussed in another section of the present review.

In 1979, the National Diabetes Data Group proposed that the diagnosis of diabetes should be established when fasting dysglicaemia levels were ≥ 140 mg/dL, and stated the condition of impaired tolerance to glucose (IGT) in those individuals submitted to OGTT presenting dysglicaemia elevation levels that ranged from ≥ 140 to <200 mg/dL 2 hours after the ingestion of dextrosol [4].

The values for diagnosing diabetes through fasting glucose have progressively changed by decreasing its value to ≥ 126 mg/dL. Another pre-diabetic condition can be diagnosed when fasting glucose is between 100 to 126 mg/dL and it has presently been named as impaired fasting glucose (IFG).

IGT and IFG, therefore, represent intermediate states of abnormal dysglicaemia and both conditions are considered pre-diabetes [5]. The pre-diabetes phase can last up to seven years in those who develop diabetes. However, a third of individuals may not progress at all and indeed a third could revert back to normal [6]. It has been estimated that, by the year of 2025, the number of people with pre-diabetes will be 472 millions [6]. Data from the World Health organization (WHO) and American Diabetes Association (ADA) estimated that around 27% of individuals with normal fasting glucose migrated to pre-diabetes and 8% to diabetes when submitted to oral glucose tolerance test (OGTT) and, moreover, 50% of subjects with dysglicemia develop diabetes [7].

The development of chronic complications of diabetes, either micro or macrovascular, can begin earlier in the pre-diabetes phase as demonstrated by UKPDS and DPP, with increasing prevalence from IFG to IGT [8,9]. The highest prevalence is observed in patients with both conditions [10]. Decreased level of HDL-cholesterol, increased level of LDL-cholesterol, triglycerides and hypertension, are present more frequently among pre-diabetic individual, increasing the cardiovascular risk [11]. Therefore, the identification of individuals with high risk for pre-diabetes must be emphasized. Some risk factors like gestational diabetes, hyperuricemia, cerebrovascular disease, peripheral disease of lower limbs, ischemic cardiopathy and polycystic ovary syndrome in women with BMI ≥ 25 kg/m^2 ^must be added to the above-mentioned. It is important to take note of the fact that many drugs have shown unfavorable effects on glucose metabolism and could induce dysdysglicaemia. Among these, antihypertensive drugs, such as betablockers and thiazidic diuretics, stand out. These drugs are widely used and recommended as first-line medications for treating hypertension [12]. It is important to highlight that such recommendations are supported by evidence from several clinical trials. For patients with ischemic cardiopathy, the use of beta-blocker is compulsory [12].

However, in modern times, the choice of antihypertensive drugs has taken into account its metabolic neutrality in addition to its effectiveness and efficacy, and for this reason, angiotensin receptor blockers (ARBs), angiotensin-converting enzyme inhibitors (ACE inhibitors) and calcium channel blockers (CCBs), either isolated or in association, have been increasingly used [13].

Concerning physiopathology, individuals with isolated IFG predominantly present hepatic insulin resistance, with normal muscle insulin resistance. The beta-cell function is impaired, with a decrease in the secretory insulin response in pool 1 (0 to 10 minutes), and pool 2 presents reduction in response at the initial stage (first 30 minutes); but at the late stage (60 to 120 minutes), this response becomes normal. However, patients with isolated IGT present normal or fairly decreased hepatic insulin sensitivity, but moderate or severe muscle resistance to the action of insulin. The beta cell presents a similar flaw, as observed in IFG patients of pool 1, i.e., decrease in insulin secretion from 0 to 10 minutes, however, with a severe insulin secretion deficit at the early stage of pool 2 [14].

## Dysglicaemia and cardiovascular disease

There is a strong relationship between dysglicaemia and cardiomorbidity and cardiomortality

### Cardiomorbidity

There is a relationship between dysglicaemia and cardiovascular atherosclerotic disease as certified by several clinical trials which have demonstrated the association of pre-diabetes with subclinical indicators of atherosclerosis, as described bellow:

1. Calcium score of coronary arteries: the average score of coronary calcification (CAC) in pre-diabetic patients is about 6.7 times increased (93 vs. 14) compared to non-diabetic individuals [15];

2. Carotid intima-media thickness: the results from the Mexico City Diabetes Study have shown that the relation of the thickness between the intima and the medium in the common and internal carotid arteries, were significantly more elevated in pre-diabetics as compared to non-diabetics [16]. European [17] and Indian studies [18] also showed the same event after adjustments to other risk factors;

3. Prevalence of cardiovascular disease: in pre-diabetics, even without metabolic syndrome, there is an increased incidence both in coronary artery disease and myocardial infarction. In patients with metabolic syndrome, such morbidities occur at a higher level, and they also present higher incidence of stroke [19];

4. Hypertension: the Framinghan study demonstrated that, for both genders, the cardiovascular event rate has been progressively increasing from the normal levels of arterial pressure. In individuals with glucose intolerance, such events also occur, though at a much higher rate, proportionally to the arterial pressure level [20];

5. Worsening of the clinical evolution of acute coronary syndromes: the *Glucose Tolerance in Patients with Myocardial Infarction *(GAMI) study [21]. Such study, performed with subjects with acute myocardial infarction, showed that only 34% of patients had normal dysglicaemia upon admittance, against 35% and 31% of pre-diabetics and diabetics, respectively. The same was observed in two other studies, the *China Heart Study (CHS) *[22]*and the Euro Heart Survey (EH*S), for which only 36% and 45% of individuals, respectively, had normal fasting dysglicaemia in the acute stage of infarction, which means that the great majority already presented a change in glucose metabolism upon admittance and were unaware of such condition;

6. Several studies have demonstrated that other biochemical markers of cardiovascular disease such as C-reactive protein, tumour necrosis factor-alpha increase in graded manner with deterioration of glucose intolerance [23];

### Cardiomortality

Studies have demonstrated the increase in the mortality rate from normal to pre-diabetic and to diabetic individuals.

1. Data from the Funagata Diabetes Study through seven years of follow-up showed that the cumulative survival rate decreases in pre-diabetics at an intermediate level between normoglycaemic and diabetic [24];

2. The Second National Health and Nutrition Examination Survey Mortality Study (NHANES 2), showed an increase both in cardiovascular and in total mortality rates, and the latter was over 40%, while comparing normoglycaemic with glucose intolerant patients [25];

3. The Collaborative Analysis of Diagnostic Criteria in Europe (DECODE), covered a broader spectrum of dysglicaemia and presents data which showed that the rate of cardiovascular death risk increases in both forms of pre-diabetes, though it is higher in IGT versus IFG [10];

4. The GAMI study showed that the free survival period of events during 50 months was significantly shorter for individuals with dysglicaemia when compared to normoglycemic patients [20];

5. The *EHS *observed that the free survival period curve of events for individuals who presented a change in glucose metabolism (impaired tolerance, recent diabetes and known diabetes) was also shorter when compared to patients with normal dysglicaemia and, again, the presence of diabetes caused a worse prognosis with survival period reduction, i.e., the longer the disease period and the more advanced its clinical stage, the shorter the cardiovascular survival period [17];

6. In conclusion, both studies (*GAMI and EHS*) showed that dysglicaemia was common in patients with coronary artery disease and it was related to a worse clinical evolution and a higher risk of other events and death.

7. *The China Heart *Study evaluated the relationship of dysglicaemia and the CV risk in the Asian population [22]. It is known that Asian populations present a difference in body fat distribution compared to Caucasians and several metabolic changes, particularly when they aggregate and compose the risk factors for metabolic syndrome, related to visceral fat. Thus, for such study, patients hospitalized with coronary artery disease, both acute and chronical, were randomized and allocated. Type 1 diabetes patients were excluded, and patients who had not been diagnosed with type 2 diabetes had taken the oral glucose tolerance test. Results showed that, in this population sample, 2/3 of patients had previous undiagnosed dysglicaemia. Currently, 80% of the individuals would have remained undiagnosed if the fasting dysglicaemia (FG) had been performed alone. Thus, similarly to the studies mentioned previously, for those individuals, fasting dysglicaemia did not reflect the real clinical setting with regards to glucose homeostasis [22]. In conclusion, *CHS *demonstrated that type 2 diabetes and pre-diabetes were also found in Asian populations with coronary artery disease, that such condition would not have been diagnosed if FPG had been performed alone and that 80% or more of those patients would have remained undiagnosed if they had not taken the OGTT.

Considering the above-mentioned data, the development of "guidelines" with recommendations for handling diabetes, pre-diabetes and CVD was performed.

The algorithm recommended by the *European Society of Cardiology *and by the *European Association for the Study of Diabetes *determines the selection of individuals in two initial groups, according to the main diagnosis, of diabetes or coronary artery disease (13). Later, strategies for the complementary diagnostic investigation for each case are suggested - so if the patient has DM 2 without clinical evidence of CAD, an investigation of the presence of coronary artery disease must be performed (electrocardiogram, echocardiogram, ergometric test and, when indicated, ischemia-stimulating tests) in order to find out if the patient originally had coronary artery disease; on the other hand, if the patient had clinical evidence of CAD, an investigation of the presence of diabetes through fasting blood glucose, OGTT and glycated haemoglobin is recommended [26].

The intensification of the concept of prevention of all forms of dysglicaemia, which started in the early 1920s, is currently recommended [27]. Several studies have been done from 1997 to 2006 emphasizing this concept. Some of them are: the Swedish Malmo Feasibility [28], the Chinese DaQing Study [29], the Finnish Diabetes Prevention (DPS) [30], the Diabetes Prevention Program (DPP) [8], the Japanese Lifestyle Intervention Study [31], the Study to Prevent Non-Insulin-Dependent Diabetes Mellitus (STOP) [32], the Troglitazone in Prevention of Diabetes (TRIPOD) [33], the Diabetes Reduction Assessment with Ramipril and Rosiglitazone Medication (DREAM) [34], among others. They reveal that treatment through lifestyle changes (LSC) and/or drug treatment decreases the conversion rate from pre-diabetic into diabetic, but the best result in the above-mentioned studies was observed with LSC.

In March 2007, the ADA proposed that all pre-diabetic patients would be recommended to undergo LSC; however, if they have combined IGT and IFG or if they are <60 years old or presented any related risk factors such as BMI ≥ 35 kg/m^2^, elevated triglycerides, reduced HDL cholesterol, hypertension, family history of diabetes and A1C >6%, metformin should be used concomitantly, at the dosage of 850 mg bid [35].

Some considerations must be discussed concerning the relationship between dysglicaemia and cardiovascular disease (CVD) and incident diabetes in developing countries like Brazil.

So far we still need data to establish which is the best predictor (fasting or post-load bood glucose or d HbA1c) of the above -mentioned clinical conditions. All these measures are related to different glycemic metabolic process [1]. Another important point is the utility of using these measures in clinical daily practice comparing to its use for epidemiological studies. The Australian Diabetes, Obesity, and Lifestyle study has used fasting and post-load glucose and HbA1c to predict CVD and all-cause mortality and whether any of these measures improved CVD and all-cause mortality beyond that achieved by traditional risk factors [36]. The study observed a linear relationship between post-load glucose and HbA1c with all-cause and CVD mortality. Fasting blood glucose showed J-shaped relationship. However none of these measures significantly improved individual risk identification over other traditional risk factors.

Another study, an observational one, with more than 40,000 members of Kaiser Permanente Northwest who had fasting plasma glucose levels less than 100 mg/dL showed an increase of diabetes risk of 6% for each mg/dL of fasting blood glucose even after controlling for other risk factors. Furthermore, increase BMI and triglycerides levels, decrease HDL-cholesterol levels presence of hypertension and smoking increase the risk of developing diabetes associated with normal fasting plasma glucose. The most important conclusion that emerged from this study was the possibility to identify high-risk patients to be screened for diabetes, thus helping clinicians in daily clinical practice [37].

Considering the overall population in Brazil (near 200 millions), the growing prevalence of diabetes, pre-diabetes and obesity [38,39] as well as the estimated increase in cardiovascular risk already observed in the population with diabetes using the Framingham equation [40] is a matter of concern. These facts highlight the importance of addressing health care and prevention issues to the high-risk subjects. All these clinical conditions must be treated intensively from its early stages. Non-pharmacological and pharmacological treatments must aim the goals for weight, glycaemic, blood pressure and lipid control. Considering pre-diabetes as a clinical entity, non-pharmacological treatment with diet and exercise gave the best results with rates of prevention of diabetes up to 60% and so must be emphasized. Pharmacological treatments are indicated when we have failure to diet and exercise with drugs which have shown clinical benefits related to reduction in morbidity and mortality. However, there is still need for new long-term studies to assess the real benefits of several therapeutical approaches, as well as its cost effectiveness.

## Competing interests

The authors declare that they have no competing interests.

## Authors' contributions

MEM: has written the cardiologist opinion.

BAC: has written the endocrinologist opinion.

SC: has written the endocrinologist opinion.

All authors have read and approved the final manuscript.
